# Artificial Intelligence in Neuro-Ophthalmology: Opportunities for the Diagnosis of Optic Neuropathies and Visual Pathway Disorders

**DOI:** 10.7759/cureus.90142

**Published:** 2025-08-15

**Authors:** Samendra Karkhur, Arushi Beri, Vidhya Verma, Saroj Gupta, Priti Singh

**Affiliations:** 1 Ophthalmology, All India Institute of Medical Sciences, Bhopal, Bhopal, IND

**Keywords:** artificial intelligence, deep learning, ischemic optic neuropathy, neuro‑ophthalmology, optical coherence tomography, optic neuritis, visual field

## Abstract

Artificial intelligence (AI) is increasingly transforming the landscape of neuro-ophthalmology by enabling earlier and more precise identification of optic nerve and visual pathway disorders. With the growing complexity of multimodal diagnostic imaging and functional assessments, AI offers a scalable solution to enhance diagnostic accuracy and streamline clinical workflows. Recent advancements, particularly in deep learning (DL) and convolutional neural networks (CNNs), have shown notable potential in interpreting fundus photography, optical coherence tomography (OCT), and magnetic resonance imaging (MRI), facilitating the detection of conditions such as optic neuritis (ON), ischemic optic neuropathy, papilledema, and glaucomatous optic nerve damage. In parallel, AI-driven analysis of visual field (VF) tests has demonstrated improved consistency in assessing disease progression, supporting longitudinal monitoring. The development of mobile diagnostic applications and integrated decision-support systems further extends the utility of AI, particularly in settings with limited specialist access. Despite these promising innovations, critical challenges remain. These include data heterogeneity across populations and imaging platforms, the opaque nature of many AI models, which limits clinical interpretability, and the absence of standardized regulatory and ethical guidelines. As the field moves toward broader clinical adoption, success will depend on robust multicenter validation studies, the creation of explainable AI (XAI) frameworks, and the implementation of strong governance structures to ensure safety, fairness, and accountability in patient care.

## Introduction and background

Artificial intelligence (AI) in neuro-ophthalmology

AI is increasingly transforming the manner in which neuro-ophthalmologists diagnose and treat disease, providing potential for earlier detection and higher accuracy of optic neuropathies and visual pathway disorders. Fundamentally, AI denotes computer systems capable of learning patterns from information and predicting or making decisions. One branch, machine learning, relies on algorithms trained on labeled examples, while deep learning (DL) - a more advanced form - uses multi-layered artificial neural networks to automatically identify complex patterns [1]. In medical imaging, convolutional neural networks (CNNs) are particularly good at making sense of spatial data, such as fundus images and optical coherence tomography (OCT) scans, whereas newer transformer models are showing great potential for the analysis of sequential or time-series data, like trends in visual fields (VFs) over time or longitudinal patient data [2].

AI is already proving clinically ready in general ophthalmology, with U.S. FDA-approved autonomous diabetic retinopathy screening systems establishing a valuable precedent [3]. These technologies open the door to equivalents in neuro-ophthalmology, where diagnosis can be particularly difficult, with subtle presentations and simultaneous imaging features. Misdiagnosis is currently a serious issue, with error rates ranging from 15% to 38%, resulting in delays to treatment and, occasionally, very significant medicolegal consequences [4]. AI, which is trained on large, well-annotated datasets, can pick up on subtle structural or vascular alterations that elude even experienced clinicians, aiding both early diagnosis and accurate tracking of disease evolution.

In the interpretation of images, CNNs have achieved expert levels of performance in detecting early papilledema and glaucomatous injury on OCT imaging - alterations most commonly missed by standard clinical observation. Fundus photograph models have demonstrated excellent accuracy in differentiating typical from atypical optic neuritis (ON), and DL in OCT angiography has surpassed standard measures in distinguishing ischemic optic neuropathy from glaucomatous optic atrophy [5]. Outside of structural imaging, AI-powered VF interpretation tools are able to detect progression earlier than clinical evaluation in isolation. In neuroimaging, radiomic analyses supported by AI of orbital and brain magnetic resonance imaging (MRI) are assisting in the differentiation between inflammatory and compressive lesions, and multimodal AI systems are being researched for the diagnosis of complicated disorders like neuromyelitis optica spectrum disorder (NMOSD) [6]. The technologies are also being incorporated into smartphone-based and decision-support applications to bring expert-level interpretation to primary care and underserved communities, where specialist access is restricted [7].

Although these advances are promising, much work lies ahead before AI can be widely integrated into routine neuro-ophthalmic practice. Most current models use narrow or homogeneous datasets, which calls into question their reliability in varied populations [8]. The "black-box" character of DL can constrain clinician trust, and the lack of standardized, externally validated trials in a range of diverse clinical environments is an indication for more stringent, real-world testing. Regulatory management, ethical protection, and the creation of explainable AI (XAI) systems will be critical to guarantee safety, fairness, and transparency in clinical take-up [9,10].

This review discusses existing and future uses of AI in neuro-ophthalmology, the clinical potential they hold, and the technical and ethical issues that need to be overcome. It is written for a general audience of neuro-ophthalmologists, general ophthalmologists, neurologists, primary and emergency care physicians, and medical AI researchers, and seeks to offer a clear introduction to the technology, alongside a critical evaluation of its influence on the future direction of the specialty.

## Review

Overview of AI in medicine and ophthalmology

AI involves a range of computational paradigms, such as supervised learning, unsupervised learning, and reinforcement learning. Of these, DL - more specifically, CNNs - has received major interest because it can handle high-volume and complex imaging data with high diagnostic precision. Whereas CNNs are largely applied to spatial data like fundus and OCT images, transformer models have proven to be useful tools for sequential and temporal analysis of datasets, such as VF progression or electronic health records [11].

In eye medicine, the notable milestone was the U.S. FDA approval of independent AI algorithms for diabetic retinopathy detection, representing a paradigm shift in clinic diagnostics [12]. The achievement has precipitated related forays in neuro-ophthalmology. Rare optic neuropathies pose special challenges for AI implementation - chiefly because of scarce annotated data, as well as heterogeneity of disease presentation.

To overcome these obstacles, methods like transfer learning and federated learning have been used. Transfer learning enables pre-trained models to be adapted to smaller, domain-related datasets, enhancing performance where data shortage is a problem. Federated learning makes it possible to utilize decentralized data sources, safeguarding patient confidentiality while aggregating clinical insights across institutions. In addition, data augmentation methods are employed to mimic variegated clinical cases, thus making model training more resilient [13]. These combined advances are broadening the scope and credibility of AI-driven applications in neuro-ophthalmology.

Current applications of AI in neuro-ophthalmology

Imaging Interpretation

Computational, machine learning-based interpretation of ophthalmic imaging has come to transform neuro-ophthalmic diagnosis. CNNs have shown unparalleled capability in differentiating genuine optic disc edema from pseudopapilledema - a differential that even experienced clinicians find challenging. Diagnostic performance has been claimed to be above 94%, especially when models are trained on high-quality fundus image datasets.

In OCT, models based on DL have been used to identify early retinal nerve fiber layer (RNFL) and macular ganglion cell-inner plexiform layer (GCIPL) alterations, making it possible to diagnose glaucoma and papilledema earlier. These features enable clinicians to pick up subtle pathological alterations that could be unrecognizable using conventional methods [14].

The combination of AI and OCT angiography has also facilitated the distinction between ischemic and glaucomatous optic neuropathies. Quantitative parameters obtained from microvasculature analysis enhance the sensitivity and specificity of diagnoses, especially in cases of overlapping clinical presentation [15].

Differentiating Optic Neuropathies

Clinical differentiation between ON and non-arteritic anterior ischemic optic neuropathy (NAION) can be tricky because of their similar features. AI models, which have been trained on multimodal imaging inputs - integrated OCT, fundus photography, and clinical factors - have produced diagnostic performances up to 93% in differentiating these entities.

In inherited optic neuropathies, like Leber's Hereditary Optic Neuropathy (LHON) and Dominant Optic Atrophy (DOA), AI models now enable early detection via superior texture and shape pattern recognition that outshines traditional interpretation.

In addition, the use of multimodal DL platforms, where ultrawide-field fundus images are merged with serological and clinical biomarkers, has been proven to identify intricate inflammatory conditions like NMOSD. Axial CNNs, fusing OCT and fundus information, have achieved 93% accuracy in differentiating between ON and NAION, whereas multimodal systems have considerably improved NMOSD classification [16-18]. 

VF Analysis

VF testing is a crucial technique in neuro-ophthalmology, but is frequently impaired by subjectivity and interobserver variation. AI algorithms have been trained to independently analyze raw Humphrey VF data and detect disease-specific patterns, with classification accuracy between 89% and 97%. Notably, transformer-based models - ideal for sequential data - are pushing the analysis of VF progression, providing clinicians with prognostic tools to track disease course and treatment response [19].

Role of AI in medicine and ophthalmology

AI is a fast-developing area, consisting of several techniques like supervised and unsupervised learning, and reinforcement learning. Of these, DL - particularly CNNs - has been especially popular because of its ability to analyze vast amounts of intricate data with unparalleled precision. CNNs have been extremely successful in reading imaging data, and newer transformer-based architectures are emerging for their potential to read sequential or time-series data, e.g., fluctuations in VFs or longitudinal patient data [11].

In ophthalmology, a landmark moment was the U.S. FDA’s approval of AI-based tools to detect diabetic retinopathy. This approval not only validated AI’s clinical potential but also inspired the expansion of AI applications into other subspecialties, including neuro-ophthalmology [12]. However, applying AI to rare optic nerve diseases remains a challenge. These conditions often lack large, well-annotated datasets - essential for training robust machine learning models.

To surpass this, various strategies have been adopted by researchers. Transfer learning enables AI models developed on large, general datasets to be fine-tuned for neuro-ophthalmic diseases using comparatively smaller datasets. Federated learning goes one step further by enabling the use of data from many institutions to train the model together - without infringing on patient privacy, as the data never leaves the original institution. In addition, data augmentation methods are employed to artificially expand datasets by generating realistic changes, enhancing the model’s ability to generalize across a wide range of clinical situations [13]. Overall, these developments are making AI instruments more viable and useful in the real-life diagnosis and care of uncommon neuro-ophthalmic conditions.

Current applications of AI in neuro-ophthalmology

Imaging Interpretation

The most compelling advancement in AI for neuro-ophthalmology remains diagnostic imaging interpretation. Fundus photography continues to serve as the primary modality, and CNNs have demonstrated exceptional performance in distinguishing true optic disc edema from pseudopapilledema - a task that proves challenging even for seasoned clinicians. Accuracy exceeding 94% has been reported in models trained on extensive datasets. Notably, hybrid models such as WHA-Net have achieved exceptional diagnostic reliability, identifying normal optic discs, optic disc edema, and pseudopapilledema with approximately 97.8% accuracy, 95.6% precision, 95.7% recall, and 98.5% specificity. Additionally, large-scale efforts such as the BONSAI (Brain and Optic Nerve Study with Artificial Intelligence) DL system have reliably distinguished papilledema from normal optic discs in non-mydriatic photographs, reinforcing AI’s potential in real-world clinical settings.

In OCT, DL algorithms are now capable of examining tiny changes in the peripapillary RNFL and macular GCIPL. Such devices are helpful in early glaucoma and papilledema detection, often before visible changes are detected on clinical examination [14]. Perhaps the most recent advancement has been the integration of OCT angiography and AI. By examining the microvascular morphology of the optic nerve head, these systems can detect glaucomatous from ischemic optic neuropathies with varying levels of accuracy. For instance, some models have achieved 91% sensitivity in diagnosing increased intracranial pressure and 95% specificity in discriminating between NAION and glaucoma. Optic disc edema arises from diverse ocular and systemic conditions, requiring timely diagnosis to prevent vision loss. Advances in multimodal imaging and AI offer improved detection, differentiation, and grading, particularly in non-ophthalmic care settings [15].

Optic Neuropathies Differentiation

Discrimination between various types of optic neuropathies remains a clinical challenge, especially when ON and NAION present with overlapping characteristics. Under these conditions, AI algorithms trained on combined datasets of OCT scans, fundus photographs, and clinical parameters have shown improved diagnostic performance, up to 93%. Inherited optic neuropathies, such as LHON and DOA, also benefit from AI processing. Texture- and shape-based pattern recognition models are able to detect earlier, more subtle changes that may be missed on routine clinical inspection. Further, AI applications are being researched for diagnosing complex inflammatory disorders such as NMOSD. Multimodal DL systems that incorporate fundus imaging, OCT information, and serological markers have enhanced diagnostic accuracy. For example, axial CNNs combining fundus and OCT information have accurately differentiated acute ON from NAION in 93% of cases, and multimodal systems have effectively boosted NMOSD detection [16-18].

Analysis of VF

VF testing is a mainstay of neuro-ophthalmic examination, but reporting these findings takes time and can vary between clinicians. AI algorithms now automatically process raw data from Humphrey VF tests to recognize typical disease patterns, with 89%-97% accuracy in classification.

Of particular interest is the use of transformer models, which have the capacity to manage sequential data. Progression trends in VF loss over time can be analyzed by these models, with the potential to provide clinicians with earlier and more accurate information about disease progression and response to treatment [19]. Table 1 shows a summary of AI applications in neuro-ophthalmology by modality.

**Table 1:** Summary of AI Applications in Neuro-Ophthalmology by Modality Visual field testing refers to automated perimetry, using threshold-based strategies such as the Humphrey Field Analyzer 24-2 or 30-2. AI, Artificial Intelligence; OCT, Optical Coherence Tomography; OCT-A, Optical Coherence Tomography Angiography; AUC, Area Under the Curve; ON, Optic Neuritis; NAION, Non-arteritic Anterior Ischemic Optic Neuropathy; MRI, Magnetic Resonance Imaging; CT, Computed Tomography

Modality	AI Application	Diagnostic Target	Performance	Reference
Fundus Photography	Disc edema vs. pseudopapilledema	Papilledema	94% accuracy, AUC 0.97	Bouthour et al. [15]
Fundus Photo + OCT	ON vs. NAION classification	Optic Neuritis, NAION	93% accuracy	Liu et al. [16]
OCT + OCT-A	NAION vs. Glaucoma differentiation	Ischemic ON, Glaucoma	95% accuracy, κ = 0.92	Bunod et al. [8]
Visual Field	Progression detection	Glaucoma, Chiasmal Defects	89%-97% accuracy	Abdalgadir et al. [9]
MRI/CT + DL	Lesion classification	Compressive vs. Demyelinating Lesions	AUC ≥ 0.90	Chiang et al. [7]
Multimodal + App	Differential diagnosis tool	Optic Neuropathies (Varied)	Diagnosis time 35%, κ = 0.82	Vinny et al. [11]

Diagnostic decision support and multimodal AI

In addition to single-modality imaging interpretation, perhaps the most revolutionary contribution of AI is its capability to deliver integrated diagnostic decision support. Multimodal AI platforms are currently emerging, integrating fundus photography, OCT scans, VF information, and clinical metadata to mimic the reasoning process of experienced clinicians. They are especially useful in difficult or uncertain cases with challenging differential diagnoses. Gradient-boosted decision models, combining clinical history, demographic information, OCT measurements, and VF indices, have provided top-three differential diagnostic accuracy rates of up to 90%. These systems are increasingly used within resource-poor environments and small clinics, where access to subspecialist care is limited. In addition, mobile and cloud-based systems are bringing diagnostic tools within reach, providing expert-grade assessments through smartphones and remote workstations. Furthermore, AI is progressing in the diagnosis of inflammatory diseases like NMOSD. DL algorithms that combine retinal imaging data with serological and clinical information have been able to identify early, subtle signs of such diseases, potentially enabling early treatment before severe visual loss is initiated [19].

Benefits of the integration of AI

Adding AI to neuro-ophthalmology has a number of concrete benefits - both in terms of enhancing diagnostic accuracy and making operations more efficient.

Of particular value among these is their capacity to recognize subclinical or preclinical disease changes. AI algorithms have the capability of detecting small changes in the optic nerve, like early thinning of the RNFL or the small elevation that is indicative of early papilledema, far ahead of the onset of symptoms or overt clinical findings. This early detection capability greatly enhances the possibility of timely intervention and improved patient outcomes. AI also improves diagnostic consistency. By applying uniform criteria to cases, it minimizes inter-observer variability - a problem that often plagues neuro-ophthalmic examinations. In addition, automated pre-screening of imaging and VF data speeds workflow, enabling clinicians to fast-track difficult cases and optimize patient care. Notably, AI tools are enabling tele-neuro-ophthalmology expansion. With smartphone-compatible imaging and cloud-based analysis platforms, diagnostic capacity can be expanded to rural and underserved areas, bridging the gaps in access to subspecialty care. Lastly, AI facilitates personalized medicine. Imaging data, combined with longitudinal clinical histories, can monitor disease courses and forecast end outcomes. This permits individualized monitoring regimens and treatment plans, mapped to each patient's specific disease trajectory [20].

Challenges and limitations

Though the potential of AI in neuro-ophthalmology is intriguing, a number of significant challenges remain to be solved before broad clinical implementation is possible. One of the main limitations is the quality, diversity, and representativeness of training datasets, along with the validity and standardization of portable imaging devices (e.g., fundus cameras, handheld scanners), and the applicability of age-related normative databases in accurately detecting disease onset and progression. Rare optic neuropathies are frequently not well represented in large datasets, risking model performance and overfitting - where the model works well on the training data but does not generalize to new, diverse patient populations. Model transparency is also a major issue. Most AI systems, especially deep-learning-based ones, are "black boxes" with little interpretability. Without insight into how these models are making decisions, clinical uptake may be slowed, as doctors will be loath to rely on systems they do not comprehend. Work is in progress to add saliency mapping and XAI techniques to render these approaches more transparent and clinically interpretable. Bias and fairness are also key concerns. Models learned from homogeneous or unrepresentative data could present performance differences in diagnostics across various demographic groups, initiating doubts about equity in care provision.

In addition, there are serious ethical and legal considerations. Matters of data privacy, informed consent, ownership of data, and medico-legal liability need to be tackled with caution. Developing strong regulatory mechanisms and ethical protocols is necessary to assure the safe, responsible, and equitable use of AI in neuro-ophthalmology [21]. Table 2 depicts the advantages and challenges of AI in neuro-ophthalmic diagnostics.

**Table 2:** Advantages and Challenges of AI in Neuro-Ophthalmic Diagnostics Table credit: [19-22] AI, Artificial Intelligence; XAI, Explainable Artificial Intelligence

Aspect	Advantages	Challenges
Diagnostic Accuracy	Early detection, high sensitivity	Model overfitting in rare conditions
Workflow Efficiency	Automated triaging, decision support	Time-intensive validation and tuning
Access and Equity	Supports tele-neuro-ophthalmology	Limited infrastructure in low-resource areas
Interpretability	XAI potential	Black-box models limit clinical trust
Data Use	Multimodal integration enhances insight	Privacy, data ownership concerns
Scalability	Fast inference once deployed	Lack of large annotated datasets

Future directions

To really unlock AI’s potential in neuro-ophthalmology, efforts in the future need to extend beyond technical enhancement and aim for significant clinical translation. The process of rendering AI models interpretable is one of the most significant steps forward. Today, most DL tools behave as "black boxes," producing outputs without explanations. Clinicians must understand why a model arrives at a conclusion - whether it is pointing to a suspicious disc on a fundus image or early glaucomatous change on OCT. That is where XAI enters. Saliency maps and attention heatmaps are techniques that can indicate what features the model looked at, and this allows clinicians to trust and act upon the outcome.

Another exciting advancement is federated learning, through which institutions can jointly train AI models without exchanging patient data. This is particularly beneficial in healthcare, where there is great importance on data privacy and rare instances are spread across centers. Federated learning enables models to learn from a broader pool of patients - enhancing accuracy, generalizability, and fairness - while not shattering confidentiality.

We also must diversify the sources of data that AI learns from. Most of today's instruments are aimed at static imaging or VF assessment. But neuro-ophthalmic conditions are multifactorial and complex. Incorporating genetic data, protein markers, or even live data from wearable sensors could provide AI with a more detailed picture - enabling personalization of treatment. Consider an AI platform that not only identifies damage but also forecasts who will decline, when, and how to treat them. All of this is irrelevant unless these tools perform in the real world. That is why we require prospective, multicenter trials to test AI models in routine practice - not merely on perfect datasets. Trials need to be based on outcomes that matter to patients, such as quicker diagnosis, fewer missed referrals, or improvement in vision over time - not merely measurements such as sensitivity or specificity [22].

Last but not least, as AI is increasingly integrated into clinical practice, we need to construct the proper ethical and regulatory guardrails. Who’s accountable if an algorithm makes a mistake? How do we prevent AI from scaling up existing healthcare inequities? Policymakers, researchers, and clinicians must collaborate to determine the answers. Meanwhile, medical education must adapt - next-generation neuro-ophthalmologists need to be as adept at interpreting AI reports as they are at reading OCTs.

## Conclusions

AI is transforming the field of neuro-ophthalmology at an ever-increasing pace, with revolutionary promise along the diagnostic spectrum. By optimizing complex imaging modality interpretation, elevating the sensitivity of function tests like visual fields, and combining heterogeneous clinical data streams, AI-powered tools have been shown to enhance diagnostic accuracy, minimize inter-observer variability, and enable earlier disease detection. In addition, these technologies facilitate triaging of high-risk cases, allowing more rapid referral to specialists and minimizing delays in diagnosis - especially in under-resourced or resource-poor environments. With inclusion in longitudinal clinical information, AI can even more enhance personalized prognostication and treatment planning, a significant step toward precision neuro-ophthalmic care. For effective translation of these innovations from development to deployment in clinical practice, however, a coordinated, multidisciplinary effort is required. Clinicians must be engaged in model development and validation to maintain the clinical relevance and credibility of outputs. Data scientists play a crucial role in constructing robust, explainable models that reflect the heterogeneity and richness of real-world patient groups. Industry players contribute by driving innovation, scaling up technologies, and integrating AI solutions into existing health infrastructure. Regulatory authorities, however, play a critical role in setting standards for validation, interpretability, safety, and ethics. In order to roll out fairly and ethically, it is also essential that the social, legal, and ethical implications of AI uptake are managed. This involves policy-making for patient confidentiality, rights of data ownership, and assigning accountability in decisions made using AI. It is only through such a multi-stakeholder and integrated approach that the potential of AI in neuro-ophthalmology can be optimally delivered - translating algorithmic innovation into measurable, quantifiable improvements in patient outcomes.
